# Effect of *Helicobacter pylori* eradication on remnant stomach neoplasms after curative gastrectomy (HELP-GC): Protocol of a HELP-GC randomized controlled trial

**DOI:** 10.1371/journal.pone.0320903

**Published:** 2025-05-19

**Authors:** Chang Seok Ko, Jin Hee Noh, Young Soo Park, Jeong Hwan Yook, Hwoon-Yong Jung, In-Seob Lee, Ji Yong Ahn, Ji Sung Lee

**Affiliations:** 1 Division of Gastrointestinal Surgery, Department of Surgery, Asan Medical Center, University of Ulsan College of Medicine, Seoul, Republic of Korea; 2 Department of Internal Medicine, Hallym University Sacred Heart Hospital, Hallym University College of Medicine, Anyang, Korea; 3 Department of Pathology, Asan Medical Center, University of Ulsan College of Medicine, Seoul, Republic of Korea; 4 Division of Gastroenterology, Department of Internal Medicine, Asan Medical Center, University of Ulsan College of Medicine, Seoul, Republic of Korea; 5 Department of Clinical Epidemiology and Biostatistics, Asan Medical Center, University of Ulsan College of Medicine, Seoul, Republic of Korea; University of Ilorin, NIGERIA

## Abstract

**Background:**

Limited research has examined the direct effectiveness of *Helicobacter pylori* eradication (HPE) on the remnant stomach neoplasms after curative gastrectomy. This study aims to assess whether HPE could prevent the development of gastric neoplasms in the remnant stomach after curative gastrectomy through a double-blinded, randomized controlled trial.

**Methods:**

After curative gastrectomy, patients with HP infection and pathologically proven stage 1 tumors will be enrolled and randomly assigned to eradication (n = 492) and placebo (n = 492) groups. Patients in the eradication arm will be given the eradication regimen, which will comprise 40 mg of esomeprazole, 1 g of amoxicillin, and 500 mg of clarithromycin twice a day for 14 days. The primary endpoint is the development of gastric neoplasms, including adenoma or adenocarcinoma. The secondary endpoints are the 10-year overall survival, improvement rates of gastric glandular atrophy and/or intestinal metaplasia, and incidence of new-onset hyperplastic polyps among the groups.

**Significance:**

This research will be the first randomized controlled clinical study in which a thorough long-term follow-up will be needed to evaluate the effectiveness of HPE for remnant stomach neoplasms after curative gastrectomy. Its results will serve as a basis for developing future strategies in the management of patients with HP infection who undergo curative gastrectomy.

**Trial registration:**

https://cris.nih.go.kr/ KCT0008855. October 10, 2023.

## Introduction

The causal relationship between *Helicobacter pylori* (HP) infection and the development of gastric cancer is well established. Previous studies reported the preventive effect of HP eradication (HPE) on the occurrence of gastric cancer [1–4]. Furthermore, HPE therapy has successfully decreased the risk of recurrent gastric neoplasms after endoscopic resection. Specifically, compared with untreated patients, patients treated with an eradication regimen experience 35%–50% reduction in the incidence of metachronous gastric adenoma and/or adenocarcinoma after endoscopic resection of the index neoplasm [5,6].

In contrast to the widely studied remarkable reduction in the incidence of gastric neoplasms after endoscopic resection but preserving entire stomach, the effect of HPE on the condition of the remaining stomach after distal or subtotal gastrectomy has been rarely investigated [7–9]. Theoretically, after surgery, the acidic condition within the stomach is inevitably changed because of alkaline reflux from small intestine, markedly reduced parietal cell volume, and denervation of vagus nerve. Based on these postoperative changes, some recent studies reported even spontaneous remission of HP in the remained stomach [10–14]. On the contrary, a previous study showed the potential role of eradication therapy after distal gastrectomy, which can improve precancerous lesions, such as glandular atrophy or intestinal metaplasia, in the remnant stomach [7]. Another study reported that the HPE rate of patients who underwent subtotal gastrectomy is similar to that of patients who did not undergo surgery, and the atrophy and intestinal metaplasia scores were significantly lower in the successful eradication group than in the other group [9]. On the basis of these findings, both studies recommended HPE after subtotal gastrectomy. In addition, a large retrospective study demonstrated remarkable prognostic benefits, namely, overall and gastric cancer-specific survival, in patients who received HPE therapy compared with those who did not have the same treatment [8]. However, studies have yet to elucidate whether HPE can effectively and directly prevent the occurrence of remnant stomach neoplasms after curative gastrectomy to provide high-level evidence. Consequently, in the patient group who receive surgery, the necessity of HPE was not proven and included in the current treatment guideline. To fill this gap, we aim to determine whether HPE can reduce the development of gastric neoplasms occurring in the remnant stomach after curative gastrectomy through a double-blinded, randomized controlled trial (RCT).

## Materials and methods

**Fig 1:**
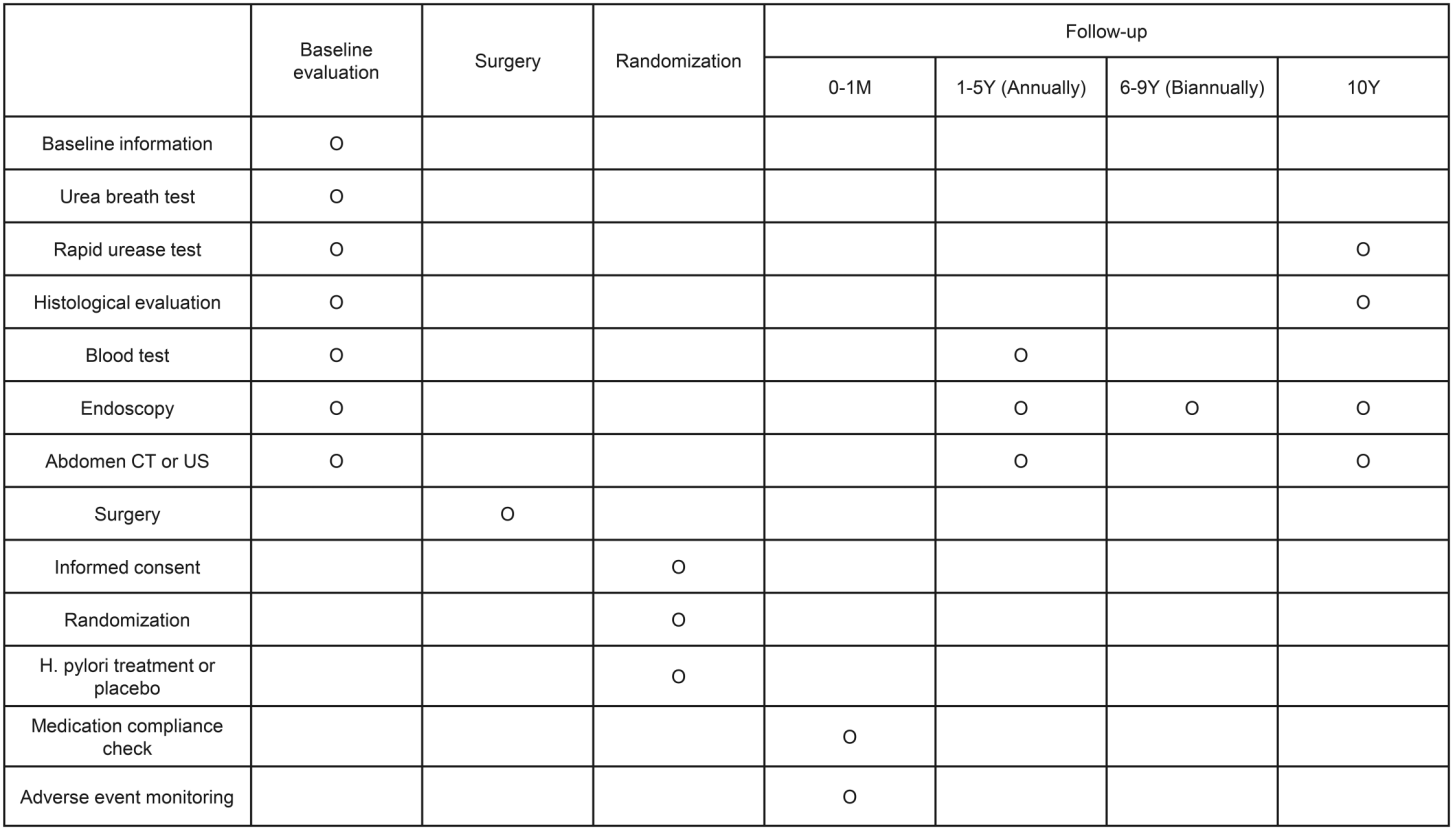
Schedule of the study.

This clinical trial is a single-center investigator-initiated, RCT that aims to analyse the effect of HPE on remnant stomach neoplasms after curative gastrectomy. The study will be conducted at Asan Medical Center, Seoul. Korea. It is scheduled to begin on June 27, 2024, and is expected to conclude on December 31, 2038. Participant recruitment will be completed by December 31, 2028. The final patient follow-up and data collection are expected to conclude by December 31, 2038. The study results are anticipated to be available by June 30, 2039. The status of this trial is now participant recruitment. Fig 1 summarizes the schedule of this trial.

### Objectives

1. Primary objective.
2. This study aims to evaluate the effect of HPE on the occurrence of gastric neoplasms, including adenoma or adenocarcinoma, in the remnant stomach of patients who undergo curative gastrectomy for gastric cancer. The follow-up period for assessing these outcomes will be 10 years.
3. Secondary objectives.
4. This study aims to examine the following: 1) 10-year overall survival, 2) improvement rates of gastric glandular atrophy and/or intestinal metaplasia, and 3) incidence of new-onset hyperplastic polyps among the groups.

### Study protocol

1 Baseline evaluation.
  1) All patients will undergo a history taking to determine whether they have previously received HPE therapy. The following tests will be performed to verify whether the patient had current HP infection prior to surgery: urea breath test, rapid urease test (RUT), and histological evaluation with Giemsa staining or immunohistochemistry (IHC).

**Fig 2:**
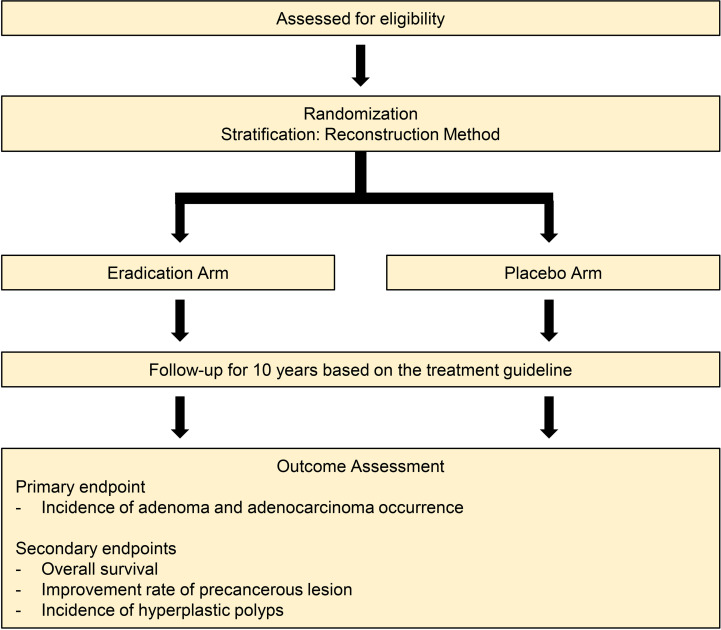
Schematic diagram of the study protocol.

- HP infection will be diagnosed when any of the three studies showed positive results.
- All patients will be asked to take a 100 mg tablet of ^13^C-urea (UBiTkit; Otsuka Pharmaceutical Co., Ltd., Tokyo, Japan) with 100 mL of water after 4 h of fasting. Two breath samples will be collected before and 20 min after the patients swallow the tablet. A cut-off value of ^13^CO_2_ < 2.5% will be considered HP negative [15].

2) Gastric glandular atrophy and intestinal metaplasia, which are known precancerous conditions, will be evaluated in biopsy specimens obtained from the gastric antrum, body, and fundus by using the updated Sydney system. Serum pepsinogen I and II concentrations and pepsinogen I/II ratio will also be assessed.

1. Surgery.
2. The patients will be subjected to curative gastrectomy with D1+ or D2 lymph node dissection based on clinical or surgical staging. Surgical treatment will be performed according to the gastric cancer treatment guidelines of Korea and Japan [16,17]. Reconstructive procedures will be selected depending on the surgeon’s preference.
3. Patient enrollment.
4. At the first outpatient clinic visit after surgery (3–4 weeks after surgery), consecutive patients with HP infection and pathologically proven stage 1 tumors will be enrolled in accordance with the following criteria for patient eligibility. Fig 2 presents a schematic diagram of the study protocol.
  - The pathological stage will be determined on the basis of the results of the histopathological examination of the resected specimen. The depths of invasion (T stage) and lymph node involvement (N stage) of tumor cells will be evaluated according to the 8th edition of the International Union Against Cancer/American Joint Committee on Cancer Staging manual.
  - Surgical curability is determined when pathological findings confirm the absence of residual tumor and an R0 resection has been achieved during the procedure.

#### Eligibility.

The following patient inclusion and exclusion criteria will be considered:

#### Inclusion criteria.

Patients will be considered eligible if they are adult patients (a) aged 19–70 years, (b) who are diagnosed with HP infection (c) diagnosed with pathologic stage 1 gastric cancer, (d) subjected to distal gastrectomy for cancer located in the lower half of the stomach (pylorus, antrum, and lower body), and (e) subjected to proximal gastrectomy for cancer located in the upper half of the stomach (fundus, cardia, upper body, and mid body) requiring either upfront curative gastrectomy or additional surgery after noncurative endoscopic resection.

#### Exclusion criteria.

Patients will be excluded if they have the following:

1) history of HPE
2) history of previous gastrectomy
3) history of any malignancy within the last 10 years
4) Patient who previously underwent endoscopic treatment on the section that will become the remnant stomach
5) patients who require adjuvant/neoadjuvant chemotherapy
6) history of allergy or serious adverse events to prescribed medication, including amoxicillin and clarithromycin
7) presence of severe comorbidities (e.g., cardiac, hepatic, or renal insufficiency) or coagulopathy
8) pregnant or lactating women
9) presence of a psychiatric disorder that might preclude compliance
10) patients who could not provide informed consent
11) Borrmann type 4 tumors (linitis plastica) on tumor classification
12) proximal resection margin shorter than 3 cm in advanced gastric cancer
13) withdrawn consent
4 Randomization.

Patients will be randomly assigned to one of the two arms in a 1:1 fashion. After the stratification of gastrointestinal reconstruction (distal gastrectomy with Billroth 1 [gastroduodenostomy] or Billroth 2 [gastrojejunostomy] or Roux-en-Y gastrojejunostomy and proximal gastrectomy with double tract reconstruction) methods, centralised stratified block randomization will be used to randomly assign the patients to the two groups.

We have performed a stratified block randomization method using a permuted block design. Stratified randomization is achieved by generating a separate block for each stratification factors, and patients are assigned to the appropriate block of stratification factors. After all patients have been identified and assigned into blocks, simple randomization occurs within each block to assign patients to one of the groups. Because the clinical trial is ongoing, we cannot provide the block size used.

An interactive web-response system will be used for randomization. The patients will be assigned to each treatment group according to the allocation codes provided by the random assignment program, and seeds will be assigned to the random assignment codes to enable reproducibility.

Double-blinding will be applied to patients and investigators. Regarding drug administration, the site investigators will check the assigned randomized drug number in order of the serial number, prepare the drug, and administer it to the patients. All the data collected from each individual patient and from the individual blinding codes will not be broken for preplanned data analysis until the database is locked.

1. Details on treatment.
  Group A (Eradication arm)
    Patients in the eradication arm will receive the eradication regimen consisting of 40 mg of esomeprazole (a proton pump inhibitor), 1 g of amoxicillin, and 500 mg of clarithromycin twice a day for 14 days.
  Group B (Placebo arm)
    Patients in the placebo arm will receive placebo tablets that are identical in appearance to the active drugs used in the eradication regimen. This ensures that both the patients and the investigators remain blinded to the treatment allocation.

All Patients will be provided with medication logs to record the administration of either the active drugs or placebos. These logs will be reviewed during follow-up visits.

1. Follow-up strategy.
  - In accordance with the Korean gastric cancer treatment guidelines, patients will be followed up with physical examination, blood tests, endoscopic examination, and imaging tests (either abdominopelvic computed tomography or ultrasonogram) at least annually for the first 5 years [16]. For the next 5 years, patients will undergo endoscopic examination biannually (Fig 1).
  - The results of HPE will be evaluated at the study endpoint using the RUT and histologic evaluation with Giemsa staining or IHC. H. pylori infection status will be considered negative only if both tests yield negative results [18].

## Outcome measurements

### Primary endpoint

#### Incidence of adenoma and adenocarcinoma occurrence.

The incidence of adenoma or adenocarcinoma will be evaluated via endoscopy and confirmed by pathologically examining the corresponding lesion. Adenomas included in the analysis will encompass both low-grade and high-grade dysplasia. Once the development of adenoma or adenocarcinoma is identified, the investigators will be required to report the occurrence of the disease by recording this information in the eCRF without delay.

### Secondary endpoints

#### Overall survival.

All deaths of the enrolled patients during the trial will be reported to evaluate overall survival.

#### Improvement rate of precancerous lesions.

Biopsy specimens will be obtained from three sites, namely, the fundus and greater curvature of the body in the remnant stomach and the anastomosis site, to assess gastric glandular atrophy and intestinal metaplasia. These parameters will be evaluated according to the updated Sydney system and compared with the scores assessed before surgery.

#### Incidence of hyperplastic polyps.

The incidence of hyperplastic polyps will be examined through endoscopy and biopsy. Once the development of a hyperplastic polyp is identified, the investigators will have to report its occurrence by recording this information on the eCRF without delay.

### Sample size calculation

On the basis of previous studies, we assumed that the 10-year incidence of adenoma and adenocarcinoma in the control group is approximately 10% [5,19]. The incidence of adenoma and adenocarcinoma will decrease by 50% after *H. pylori* eradication. According to this assumption, if the primary endpoint is exponentially distributed in both groups, the expected hazard ratio (HR) for the treatment group is 0.49. One interim analysis will be planned using the O’Brien-Fleming spending function at 50% of the total primary endpoint events. A total of 66 primary endpoint events will yield approximately 80% power to detect a HR of 0.49 with an overall type I error of 0.05 (two sided). Considering a 10% dropout rate, 984 randomized patients will be enrolled. The sample size will be calculated using PASS 15 software (NCSS, LLC, Kaysville, Utah, USA).

### Interim analysis

One interim efficacy analysis will be planned for the primary endpoint by using the O’Brien-Fleming spending function at 50% of the total primary endpoint events. The primary endpoint will be tested at a two-sided nominal type I error of 0.003. The final efficacy analysis will be performed after approximately 66 primary endpoint events are observed. The primary endpoint will be tested at a two-sided nominal type I error of 0.049. The alpha spending for interim and final analyses based on the planned number of primary endpoint events is summarised in Table 1. The actual alpha spending will be based on the actual number of primary endpoint events included in the analyses and determined by the O’Brien-Fleming spending function at the time of interim and final analyses.

**Table 1:** Alpha spending in group sequential design using the O’Brien Fleming spending function for the primary endpoint.

First Interim Efficacy Analysis		Final Efficacy Analysis	
# Events needed	2-sided Nominal Alpha	# Events needed	2-sided Nominal Alpha
33	0.003	66	0.049

### Data management and monitoring

Data will be collected using a web-based eCRF developed by Procuratio®. The eCRFs will be completed at study enrollment and during follow-up outpatient visits. This system will anonymise and secure all data. Authorized staff members will enter the data into the electronic database, ensuring it is accurate and complete. This trial will be monitored by an independent data and safety monitoring board (DSMB) organized by independent experts, including a biostatistician.

### Safety monitoring

Adverse events (AEs) and serious adverse events (SAEs) will be documented from the moment the patient signs the informed consent form until the follow-up period ends. The investigator must notify the Ethics Committee and/or the Regulatory Authority of any AEs or SAEs, as per local regulations.

### Statistical analysis

#### General considerations.

The full details of the statistical analyses will be described in the Statistical Analysis Plan (SAP), which will be finalised prior to code breaking. Analyses will be performed using SAS software (SAS Institute, Inc., Cary, North Carolina, USA); The version used will be specified in the SAP. Categorical data will be summarised by the number and percentage of subjects in each category. Continuous variables will be summarised by descriptive statistics, including the number of observations, mean, standard deviation, median, minimum, and maximum.

We will use the Kaplan-Meier method to calculate the cumulative incidence of adenoma, adenocarcinoma, and hyperplastic polyps, as well as overall survival. These will be compared between the two treatment groups using the log-rank test. The HRs, with 95% CIs, will be estimated using a Cox proportional hazards model. The improvement rate of precancerous lesion and the eradication rate of HP will be compared between treatment groups using the Chi-square test or Fisher’s exact test.

Unless otherwise stated, all efficacy analyses (except for the primary endpoint) will be conducted with a two-sided test at a significance level of α = 0.05.

#### Population analysis.

The details of each population below and any additional population, if needed, will be described in the SAP. The full analysis set (FAS) will be used as the primary population for all efficacy analyses. Dispositional summaries, demographic data, baseline characteristics, protocol deviations, and efficacy endpoints will be utilised for this analysis. In addition, the primary analysis will be repeated using the per-protocol (PP) set.

#### FAS.

The FAS will include all randomized patients, which will constitute the intention-to-treat population. The FAS will be based on the treatment allocated (as randomized). All efficacy endpoints will be analysed using the FAS.

#### Per-protocol set.

PP set will be defined as all patients in the FAS who meet the inclusion or exclusion criteria and receive treatment according to the protocol. Patients with poor compliance, defined as taking less than 75% of the medication, will be excluded from the PP population.

#### Safety analysis set (SS).

The SS will include all randomized patients who receive any study drug. This set will be based on the treatment received (as treated). Treatment compliance/administration and all clinical safety variables will be analysed using the SS.

#### Ethics and dissemination.

After the study is approved by the institutional review board of the Asan Medical Center, Seoul, Korea, the first patient will be enrolled (IRB No. 2023–0835). This study will be performed in accordance with the Declaration of Helsinki.

Written informed consent will be obtained from all patients before they are recruited. The protocol of this trial, titled “Effect of *Helicobacter pylori* eradication on remnant stomach neoplasm after curative gastrectomy (HELP-GC),” is registered at https://cris.nih.go.kr/as KCT0008855.

The findings of this study will be disseminated through publication in peer-reviewed journals and through presentations at academic conferences.

## Discussion

This double-blinded randomized clinical trial aims to evaluate the effectiveness of HPE in remnant stomach neoplasms after curative gastrectomy. The significance of the current study is described as follows.

First, a pivotal consideration in our research is the deliberate choice of adenoma and adenocarcinoma incidence in the remnant stomach as our primary outcomes. Thus, the effectiveness of HPE in terms of lesions requiring endoscopic or surgical intervention can be directly evaluated as a primary endpoint. Previous RCTs analysed the overall survival or changes in atrophy and metaplasia as primary outcomes; as such, deriving definitive conclusions about the actual impact of HPE on neoplasms in the remnant stomach is challenging [7,9]. With the release of our study results, we will provide critical insights into this matter.

Second, a 10-year long-term follow-up study post randomization will be implemented; thus, the prolonged effects of HPE will be assessed. Tumors that arise in the remnant stomach after curative gastrectomy for gastric cancer treatment require a minimum of approximately 7 years to develop [20–24]. Thus, extended tracking is necessary to investigate remnant stomach neoplasms.

Third, we aim to optimise the randomization process by incorporating the reconstruction method as a stratification factor. This strategic approach is designed to effectively control confounding variables, promote similarity in baseline characteristics, and improve the statistical power of our analysis. Previous research reported variations in the occurrence of remnant gastric cancer associated with different reconstruction methods [19,25–27]. A previous report suggested a higher risk of remnant gastric cancer in patients who underwent proximal gastrectomy, preserving the distal stomach [28]. Therefore, we will include patients who underwent proximal gastrectomy in our protocol. This inclusion is based on our expectation that proximal gastrectomy is associated with a relatively higher likelihood of occurrence of remnant gastric cancer. By incorporating these considerations, our study aims to contribute valuable insights into the reconstruction methods and their implications for remnant gastric cancer risk.

## Conclusions

This clinical trial will be the first double-blinded randomized controlled study that will require a thorough long-term follow-up to assess the efficacy of HPE on remnant stomach neoplasms after curative gastrectomy. The results derived from this research will serve as a basis for developing future strategies in managing HP-infected patients who undergo curative gastrectomy.

## Supporting information

S1 File SPIRIT checklist. (DOC)

S2 File Study protocol. (DOCX)

S1 Data Informed consent form english. (PDF)

S2 Data CRIS english. (PDF)
