# Supplementary material for: Effect of Helicobacter pylori eradication on remnant stomach neoplasms after curative gastrectomy (HELP-GC): Protocol of a HELP-GC randomized controlled trial
Source: PLoS One. 2025 May 19;20(5):e0320903. doi: 10.1371/journal.pone.0320903 (PMC12088511; doi:10.1371/journal.pone.0320903)
Supplement: S2 File — (DOCX) [file pone.0320903.s002.docx]

**Study Protocol**

근치적 위절제술 후 잔위 신생물에 대한 헬리코박터 제균치료의 효과

Effect of *Helicobacter pylori* eradication on remnant stomach neoplasm after curative gastrectomy

Principal-Investigator: In-Seob Lee

Co-Investigator: Ji Yong Ahn

Co-Investigator: Beom Su Kim

Co-Investigator: Moon-Won Yoo

Co-Investigator: Chung Sik Gong

Co-Investigator: Chang Seok Ko

Co-Investigator: Sa Hong Min

Co-Investigator: So Young Byun

# Summary

| **Title** | (Korean) 근치적 위절제술 후 잔위 신생물에 대한 헬리코박터 제균치료의 효과  (English) Effect of *Helicobacter pylori* eradication on remnant stomach neoplasm after curative gastrectomy |  |
| --- | --- | --- |
| **Objectives** | This study aims to assess whether HPE could prevent the development of gastric neoplasms in the remnant stomach after curative gastrectomy through a double-blinded, randomized controlled trial. |  |
| **Principal Investigator** | In-Seob Lee |  |
| **Research Institution** | Division of Gastrointestinal Surgery, Department of Surgery, Asan Medical Center, University of Ulsan College of Medicine, Seoul, Republic of Korea |  |
| **Participants** | 1. **Inclusion Criteria**    1. Aged 19–70-years-old    2. Patients who are diagnosed with HP infection    3. Patients who are diagnosed with pathologic stage 1 gastric cancer    4. Subjected to distal gastrectomy for cancer located in the lower half of the stomach (pylorus, antrum, and lower body)    5. Subjected to proximal gastrectomy for cancer located in the upper half of the stomach (fundus, cardia, upper body, and mid body)    6. Requiring either upfront curative gastrectomy or additional surgery after noncurative endoscopic resection. 2. **Exclusion Criteria** 3. History of HPE 4. History of previous gastrectomy 5. History of any malignancy within the last 10 years 6. Patient who previously underwent endoscopic treatment on the section that will become the remnant stomach 7. Patients who require adjuvant/neoadjuvant chemotherapy 8. History of allergy or serious adverse events to prescribed medication, including amoxicillin and clarithromycin 9. Presence of severe comorbidities (e.g., cardiac, hepatic, or renal insufficiency) or coagulopathy 10. Pregnant or lactating women 11. Presence of a psychiatric disorder that might preclude compliance 12. Patients who could not provide informed consent 13. Borrmann type 4 tumours (linitis plastica) on tumour classification 14. Proximal resection margin shorter than 3 cm in advanced gastric cancer 15. Withdrawn consent |  |

|  |  |
| --- | --- |
| **Study Design** | Single-center, randomized, double-blind study |
| **Methods** | 1. Preoperative urea breath test (UBT) & rapid urease test (RUT), histology, Giemsa or immunohistochemistry [IHC] staining), and pepsinogen study will be conducted.   -> If at least one of the UBT, RUT, or histology tests is positive, the patient will be diagnosed with *H. pylori* infection.   1. At the first outpatient visit after gastric cancer surgery, pathologic examination and *H. pylori* infection test results are confirmed, and patients with stage I gastric cancer and *H. pylori* positivity are randomized to treatment and placebo groups. 2. Group A (Treatment arm): Esomeprazole 40 mg, amoxicillin 1 g, and clarithromycin 500 mg twice a day, total of 14 days   Group B (Placebo arm): Three placebo drugs   1. Remnant stomach neoplasm development and recurrence will be assessed for 10 years after surgery through blood tests, gastroscopy, and imaging (abdominal pelvic computed tomography or abdominal ultrasound) based on the Korean Gastric Cancer Treatment Guidelines. 2. At the end of the follow-up, RUT tests and histological examinations (Gastric glandular atrophy, intestinal metaplasia, Giemsa, or immunohistochemistry (IHC) stain) will be performed to investigate the *H*. *pylori* infection rate and assess the remission rate of precancerous lesions (glandular atrophy and intestinal metaplasia) at three locations preoperatively (antrum, body, fundus) and three locations postoperatively (fundus, greater curvature of body, anastomosis) by biopsy based on the updated Sydney system. |

| **Outcome measurements** | - Primary endpoint: Incidence of gastric adenoma or gastric cancer in the remnant stomach - Secondary endpoints: 1) Overall survival; 2) improvement rate of precancerous lesions; and 3) incidence of hyperplastic polyp |
| --- | --- |
| **Statistical Analysis** | Clinical characteristic variables will be presented as numbers and fractions, means and standard deviations, or medians, ranges, and quartiles, depending on the nature of the variable. The Chi-squared test and Fisher's exact test will be used to compare categorical variables. Student's t-test and Mann–Whitney U test will be used to compare continuous variables, depending on the nature of the variable. The correlation of each variable will be expressed as a correlation coefficient. Overall survival and disease-specific survival will be analyzed using the Kaplan–Meier method and compared using the Cox proportional hazards regression model. |

## Table of Contents

1. Cover
2. Summary
3. Background
4. Objectives
5. Study Methods
   1. Study Design
   2. Participants
   3. Participants’ Consent
   4. Methods
   5. Statistical Analysis of Data
   6. Follow-up Participant Care
6. Ethical Considerations
7. Schedules
8. References

# Background

*Helicobacter pylori* (*H. pylori*) is one of the most common infectious diseases, with prevalence rates varying by geographical region and exhibiting differences in accordance with age and race. Although the prevalence of *H. pylori* in Korea has been on a steady decline since its first discovery in 1998, according to a national survey conducted by the Korean College of Helicobacter and Upper Gastrointestinal Research in 2015, the prevalence is still high at 51.0%, with a prevalence of over 50% in people over 40-years-old. *H. pylori* infection is known to be strongly associated with peptic ulcer disease, chronic gastritis, marginal zone B cell lymphoma, and gastric cancer.

The WHO recognized H. pylori infection as a carcinogenic factor in 1994, with approximately 89% of non-branchial gastric cancers and 78% of all gastric cancers being associated with *H. pylori* infection. A previous meta-analysis reported that *H. pylori* eradication reduced the risk of gastric cancer by a factor of 0.64. Additionally, a Korean study demonstrated that *H. pylori* eradication in patients who underwent an endoscopic resection for early gastric cancer significantly reduced the rate of gastric cancer recurrence, compared to the placebo group. Therefore, it is currently recommended that *H. pylori* be eradicated in patients who have undergone endoscopic resections for early gastric cancer, to lower the incidence of secondary gastric cancer.

Conversely, there is no consistent policy on *H. pylori* eradication after gastrectomy. Following gastric cancer surgery, the colonization density of *H. pylori* is reduced due to the decreased area of stomach tissue. Additionally, the reflux of bile acids resulting from postoperative structural changes induces an inflammatory response in the gastric tissue and alters the acidity of the gastric fluid, creating a relatively unfavorable environment for *H. pylori* to survive. Therefore, the diagnosis, treatment, and follow-up of *H. pylori* infection in the remnant stomach requires a different approach to that pursued with normal healthy stomachs.

These challenges have led to a lack of research on the short- and long-term effects of *H. pylori* eradication in patients with gastric cancer who have undergone a radical gastrectomy, and existing studies have had mixed results. A prospective randomized study of 190 patients with a previous history of surgery for gastric cancer revealed a notable reduction in atrophy and intestinal epithelialization in the eradication group when compared to the placebo group at the 36-month postoperative follow-up. However, a double-blind, randomized, prospective study of 169 patients with gastric cancer from the same patient registry reported that *H. pylori* eradication did not improve long-term survival in patients who had undergone gastrectomy. A relatively recent Korean study with 1,031 patients with confirmed *H. pylori* infection who underwent surgery for gastric cancer identified a significantly higher overall survival (96.5% vs. 79.9%) and gastric cancer-specific survival (97.6% vs. 92.5%) among those who had received eradication, compared to those who had not receive eradication, or those who had failed.

Long-term follow-up is required to evaluate the effectiveness of eradication in the remnant stomach, and a long-term follow-up of five years or more at a single institution is not feasible. Taking this into account, well-designed, large-scale, long-term follow-up studies are needed, as retrospective studies are limited in establishing long-term effects. Therefore, this study aims to determine whether *H. pylori* eradication in the residual stomach has a preventive effect on the development of gastric neoplasms in the long term; to further analyze the long-term survival rate, cure rate of precancerous lesions, and success rate of postoperative *H. pylori* eradication; and assess the incidence of postoperative hyperplastic polyps.

# Objectives

4-1. The difference in the incidence of gastric adenoma or gastric cancer in the remnant stomach in patients undergoing radical gastrectomy for primary stage I gastric cancer will be compared between two groups: those with and those without *H. pylori* eradication.

4-2. Overall survival rates, improvement rates of precancerous lesions, , and the incidence of hyperplastic polyps will be compared between the two groups.

# Study Methods

## 5-1. Study Design:

- Single-center, prospective, double-blind, randomized controlled study.
- Patients with confirmed *H.* pylori infection will be randomized in a 1:1 ratio to receive eradication (antibiotics) or placebo (placebo drugs) using a program in an internet-based data manager system. Neither the investigators nor the subjects will be aware of the specific medication used in advance.

## 5-2. Participants:

1. **Inclusion Criteria**
   1. Aged 19–70-years-old
   2. Patients who are diagnosed with HP infection
   3. Patients who are diagnosed with pathologic stage 1 gastric cancer
   4. Subjected to distal gastrectomy for cancer located in the lower half of the stomach (pylorus, antrum, and lower body)
   5. Subjected to proximal gastrectomy for cancer located in the upper half of the stomach (fundus, cardia, upper body, and mid body)
   6. Requiring either upfront curative gastrectomy or additional surgery after noncurative endoscopic resection.

## Exclusion Criteria

1. History of HPE
2. History of previous gastrectomy
3. History of any malignancy within the last 10 years
4. Patient who previously underwent endoscopic treatment on the section that will become the remnant stomach
5. Patients who require adjuvant/neoadjuvant chemotherapy
6. History of allergy or serious adverse events to prescribed medication, including amoxicillin and clarithromycin
7. Presence of severe comorbidities (e.g., cardiac, hepatic, or renal insufficiency) or coagulopathy
8. Pregnant or lactating women
9. Presence of a psychiatric disorder that might preclude compliance
10. Patients who could not provide informed consent
11. Borrmann type 4 tumours (linitis plastica) on tumour classification
12. Proximal resection margin shorter than 3 cm in advanced gastric cancer

## Target Number of Participants

In light of prior research, it can be reasonably inferred that a 10-year incidence of gastric adenoma and gastric cancer of approximately 10% can be expected in the control group, with a 50% reduction in the incidence of gastric adenoma and gastric cancer following *H. pylori* eradication. Consequently, to achieve a power of 80% with a 1-sided type I error of 0.05, it will be necessary to include 441 subjects in each arm of the study. The experimental and control groups will be randomized in a 1:1 ratio, with 492 subjects in each group, considering a possible 10% dropout rate, for a total of 984 subjects.

## Discontinuation and Dropout Criteria

- 1. Request by the participant or the participant’s legal guardian to discontinue participation in the study.
  2. Determined by the investigator to be unreasonable for the participant to continue in the study.

## 5-3. Participants’ Consent

This is a prospective study, which will be conducted in accordance with the tenets of the Declaration of Helsinki. Furthermore, the protocol will be approved by the Institutional Review Board of Asan Medical Center. The purpose, methods, and possible adverse events of the study will be explained in a separate room before participation in the course of obtaining informed consent, and participants will be free to terminate their participation at any time during the course of the study. No personal information other than the patient's sex, age and variables for outcome analysis will be provided to others, and all patient information other than the variables necessary for the study will be anonymized by the principal investigator in the hospital. The same principle applies to the post-test report.

## 5-4. Methods

1. **Methods**
   - **Clinical Study Methods**

Preoperative testing will include the urea breath test (UBT), rapid urease test (RUT), histology (Glandular atrophy, Intestinal metaplasia, Giemsa, or immunohistochemistry [IHC] staining), and pepsinogen study. A positive result on at least one of the UBT, RUT, or histology tests will confirm a diagnosis of *H. pylori* infection.

At the first outpatient visit after gastric cancer surgery, a pathological examination and the *H. pylori* infection test will be performed, and patients with stage I gastric cancer and *H. pylori* positivity will be randomized to the following treatment and placebo groups.

- Group A (Treatment arm): Esomeprazole 40 mg, amoxicillin 1 g, and clarithromycin 500 mg twice a day for total of 14 days
- Group B (Placebo arm): Three placebo drugs

Remnant stomach neoplasm development and recurrence will be assessed for 10 years post-surgery through blood tests, gastroscopy, and imaging (abdominal pelvic computed tomography or abdominal ultrasound) based on the Korean Gastric Cancer Treatment Guidelines.

At the end of the follow-up, RUT tests and histological examinations (Giemsa, or IHC staining) will be performed to investigate the *H. pylori* infection rate and assess the remission rate of precancerous lesions (glandular atrophy and intestinal metaplasia) at three locations preoperatively (antrum, body, fundus) and three locations postoperatively (fundus, greater curvature of body, anastomosis) by a biopsy based on the updated Sydney system.

## Data Collection Items

1. Factors associated with the patient: sex, age, comorbidities, alcohol/smoking history, medications, history of eradication, history of digestive disease, symptoms, allergies, history of surgery, and family history.

2. Diagnostic test results for *H. pylori* infection: rapid urease test, urea breath test, histology.

3. Endoscopic findings: stomach cancer location, size, shape, and biopsy results.

1. Surgery-related assessments: surgery date, time, and method; the extent of gastric resection; anastomosis method; lymph node resection; radical resection; tumor location; blood volume and transfusion; complications; and discharge date.
   1. Pathological findings: tumor location, differentiation, Lauren classification, gross type, tumor size, number of tumors, margin involvement, T/N/M stage, and number of transitional lymph nodes.
   2. Clinical outcomes after eradication: date of medication initiation, medication adherence, medication adverse events, and success of eradication.

## 3) Efficacy Evaluation

Efficacy Evaluation Variables

-Primary endpoint: Incidence of gastric adenoma or gastric cancer in the remnant stomach

-Secondary endpoints: 1) Overall survival; 2) improvement rate of precancerous lesions; and 3) incidence of hyperplastic polyp

## Safeguards for Participant Protection

- Monitoring of safety data (adverse event monitoring).
- Stability will be assessed regularly from the time of written consent through regular follow-up visits. Information about all adverse events, whether self-reported by the patient, discovered by the investigator, or detected by physical examination, laboratory testing, or other means, will be recorded on an adverse event case report form and monitored as appropriate.

Ways to monitor and check the conduct of the study will be presented.

- A Data and Safety Monitoring Board will be established to oversee the safety of this study.
- The potential for harm from the study will be examined, and recommendations for action will be made.
- Periodic review of participant safety and study progress will be conducted.
- A specialized CRO will be selected to conduct periodic monitoring to check the progress of the study and related matters.

## 5) Statistical Analysis of Data

Clinical characteristic variables are presented as numbers and fractions, means and standard deviations, or medians, ranges, and quartiles, depending on the nature of the variable. The Chi-squared and Fisher's exact tests will be used to compare categorical variables. The Student's t-test and Mann–Whitney U test will be used to compare continuous variables, depending on the nature of the variable. The correlation of each variable will be expressed as a correlation coefficient. Overall survival and disease-specific survival will be analyzed using the Kaplan–Meier method and compared using the Cox proportional hazards regression model.

## 6) Follow-up Participant Care

After the study, patients will receive the same routine care as the general population.

## 7) Interim Analysis

One interim efficacy analysis will be planned for the primary endpoint by using the O’Brien-Fleming spending function at 50% of the total primary endpoint events. The primary endpoint will be tested at a two-sided nominal type I error of 0.003. The final efficacy analysis will be performed after approximately 66 primary endpoint events are observed. The primary endpoint will be tested at a two-sided nominal type I error of 0.049. The actual alpha spending will be based on the actual number of primary endpoint events included in the analyses and determined by the O’Brien-Fleming spending function at the time of interim and final analyses.

# Ethical Considerations

## Compensation Measures

- Gastrectomy and *H. pylori* eradication are well-established and universal treatment modalities for gastric cancer, with appropriate medical or surgical interventions for the predictable complications associated with it. However, these treatments will not be reimbursed.
- Appropriate medical attention will be given in cases of unexpected complications.

## In the event of unexpected complications resulting in serious bodily injury, birth defects, or death, clinical research insurance will be used to provide compensation.

## Support for Participants

None.

## 4) Additional Costs Incurred by Participants - None.

## Measures to Protect the Privacy of Participants

- Research materials, including case notes, will be documented, encrypted, and stored on computers with restricted access, and access to patient information will be limited to the principal investigator and co-investigators. The data will be retained for five years after the completion of this study, for future inspection. After the end of the retention period, paper documents will be shredded, and electronic documents will be permanently deleted.
- The data of subjects who have given their consent to participate in this study will be anonymized and will not be viewed, collected, or used by anyone other than those involved in the study at the collaborating institutions.

# Schedules

|  |  |  |  |  |  |  | **(Study period: From IRB approval date to** | | | | | | | **December 31,** | | **2032)** | |
| --- | --- | --- | --- | --- | --- | --- | --- | --- | --- | --- | --- | --- | --- | --- | --- | --- | --- |
| Study content / Year | | | | | **0.2** | **0.5** | **1** | **2** | **3** | **4** | **5** | **6** | **7** | | **8** | **9** | **10** |
| IRB submission | | | | |  |  |  |  |  |  |  |  |  | |  |  |  |
| Informed consent, randomization, gastric cancer treatment, *H. pylori* eradication, follow-up data collection | | | | |  |  |  |  |  |  |  |  |  | |  |  |  |
| Interim and final result analyses | | | | |  |  |  |  |  |  |  |  |  | |  |  |  |
| Writing and reporting of papers | | | | |  |  |  |  |  |  |  |  |  | |  |  |  |

1. **References**
2. Lee JH, Choi KD, Jung HY, Baik GH, Park JK, Kim SS, et al. Seroprevalence of Helicobacter pylori in Korea: A multicenter, nationwide study conducted in 2015 and 2016. Helicobacter. 2018;23(2):e12463.
3. Chen HN, Wang Z, Li X, Zhou ZG. Helicobacter pylori eradication cannot reduce the risk of gastric cancer in patients with intestinal metaplasia and dysplasia: evidence from a meta-analysis. Gastric Cancer. 2016;19(1):166-75.
4. Choi IJ, Kook MC, Kim YI, Cho SJ, Lee JY, Kim CG, et al. Helicobacter pylori Therapy for the Prevention of Metachronous Gastric Cancer. N Engl J Med. 2018;378(12):1085-95.
5. Lin YS, Chen MJ, Shih SC, Bair MJ, Fang CJ, Wang HY. Management of Helicobacter pylori infection after gastric surgery. World J Gastroenterol. 2014;20(18):5274-82.
6. Cho SJ, Choi IJ, Kook MC, Yoon H, Park S, Kim CG, et al. Randomised clinical trial: the effects of Helicobacter pylori eradication on glandular atrophy and intestinal metaplasia after subtotal gastrectomy for gastric cancer. Aliment Pharmacol Ther. 2013;38(5):477-89.
7. Kim YI, Cho SJ, Lee JY, Kim CG, Kook MC, Ryu KW, et al. Effect of Helicobacter pylori Eradication on Long-Term Survival after Distal Gastrectomy for Gastric Cancer. Cancer Res Treat. 2016;48(3):1020-9.
8. Choi Y, Kim N, Yun CY, Choi YJ, Yoon H, Shin CM, et al. Effect of Helicobacter pylori eradication after subtotal gastrectomy on the survival rate of patients with gastric cancer: follow-up for up to 15 years. Gastric Cancer. 2020;23(6):1051-63.
