# Supplementary material for: Effect of Helicobacter pylori eradication on remnant stomach neoplasms after curative gastrectomy (HELP-GC): Protocol of a HELP-GC randomized controlled trial
Source: PLoS One. 2025 May 19;20(5):e0320903. doi: 10.1371/journal.pone.0320903 (PMC12088511; doi:10.1371/journal.pone.0320903)
Supplement: S1 Data — (PDF) [file pone.0320903.s003.pdf]

Participant Information Sheet and Informed Consent Form

Study Title:

Effect of Helicobacter pylori eradication on remnant stomach neoplasms after curative gastrectomy (HELP-GC)

Subject ID:

Participant Information Sheet

You are invited to participate in this clinical research study. Before deciding whether to take part, it is important to understand the purpose of the study and what it involves. This document provides information about the study's purpose, duration, procedures, benefits, potential risks, and precautions. It also outlines your rights, including the right to withdraw from the study at any time.

Please read this information carefully and feel free to discuss it with your family or others. If you have any questions, you may contact the principal investigator or research staff at any time.

Participation in this study is voluntary and is not part of your routine medical care. By signing this form, you confirm that you have received an explanation of the study and its potential risks and that you (or your legal representative) agree to participate. You will receive a signed copy of this form for your records.

1. Study Background and Objectives

Helicobacter pylori infection is one of the most common infectious diseases worldwide, with a prevalence of approximately 50% in Korea. It is strongly associated with peptic ulcers, chronic gastritis, marginal zone B-cell lymphoma, and gastric cancer. The World Health Organization (WHO) classifies H. pylori as a carcinogen, and approximately 78% of gastric cancers are linked to H. pylori infection.

A previous study in Korea showed that eradication therapy in patients who underwent endoscopic resection for early gastric cancer significantly reduced the recurrence rate compared to a placebo group. As a result, eradication therapy is now recommended for such patients to reduce the risk of metachronous gastric cancer.

However, there is no established consensus regarding H. pylori eradication therapy after gastrectomy. Surgical changes in the stomach—such as reduced volume, bile reflux, and altered acidity—affect the diagnosis, treatment, and monitoring of H. pylori infection, and in some cases, the infection may resolve naturally. This has led to differing opinions on whether eradication therapy effectively prevents remnant gastric neoplasms, with previous studies reporting inconsistent outcomes.

To assess the effect of eradication therapy on remnant gastric neoplasms, standardized diagnostic methods and long-term follow-up are necessary. However, long-term studies involving large numbers of patients remain limited. This study aims to evaluate the long-term preventive effect of H. pylori eradication after gastrectomy on remnant gastric neoplasms and analyze its impact on long-term survival and regression of precancerous lesions.

2. Randomization to Eradication or Placebo Group

If you agree to participate, you will be randomly assigned to one of two groups:

① H. pylori eradication therapy group

② Placebo group

Assignment will be done using a computer-generated randomization process, with a 50% chance of being allocated to either group.

3. Study Procedures and Methods

1) Collection of Clinical Data

If you consent to participate, you will be asked about your medical history, current medications, alcohol and tobacco use, and family history. Additional data will be obtained from your medical records, including results from endoscopic and biopsy examinations (OLGA/OLGIM staging, Giemsa or immunohistochemistry staining), pepsinogen studies, CT scans, and laboratory tests. Post-randomization, information related to eradication therapy and follow-up assessments will also be collected.

2) Randomized Treatment Assignment

Before undergoing curative gastrectomy, blood and tissue samples will be collected during endoscopy to test for H. pylori. If infection is confirmed and you consent to participate, you will be randomly assigned to either the eradication or placebo group. The eradication group will receive the standard 14-day triple therapy (esomeprazole, amoxicillin, clarithromycin), and the placebo group will receive matching placebo tablets.

3) Follow-up Monitoring

After treatment for gastric cancer, you will receive routine follow-up evaluations to check for cancer recurrence, remnant gastric neoplasms (e.g., adenomas, polyps), or complications. You will undergo periodic blood tests, endoscopy, and imaging (CT or abdominal ultrasound) for a period of 10 years. At the end of the follow-up period, H. pylori status will be reassessed via blood test and biopsy.

If recurrence or a new lesion is detected, additional evaluations such as biopsy, endoscopic ultrasound, or PET-CT may be performed to determine the stage, and further treatment (e.g., endoscopic resection, surgery, or chemotherapy) may be administered.

4. Participant Responsibilities

During the study, you are expected to attend all scheduled visits and comply with study procedures. You must report any changes in your health to your study physician or coordinator, even if the changes are unrelated to the study. You may withdraw from the study at any time by informing the study team.

5. Participation Duration and Visit Schedule

The study is planned to continue until December 2032. Follow-up visits will be scheduled every 6 to 24 months as part of your routine post-operative care.

6. Study Population

This study will enroll 984 participants between the ages of 19 and 70 who have undergone curative gastrectomy for stage I primary gastric cancer and tested positive for H. pylori. The study will be conducted at Asan Medical Center.

7. Expected Complications, Risks, and Discomforts

Gastrectomy and H. pylori eradication therapy are standard treatments with known risks:

Surgical complications may occur in 10–20% of patients. Short-term complications (within 30 days) include bleeding, infection, atelectasis, pneumonia, respiratory failure, liver dysfunction, acute kidney injury, anastomotic leakage, and bowel obstruction. Long-term complications may include wound infections, obstructions, or strictures, which may require conservative treatment, endoscopic procedures, or additional surgery.

Eradication therapy side effects may include nausea, abdominal pain, or diarrhea. These are typically manageable, but in rare cases, more severe reactions such as skin rash may require discontinuation of antibiotics.

8. Compensation and Costs

The study will cover the cost of the final H. pylori test. You will receive confirmation of your infection status, and if positive, treatment may be recommended (treatment costs will be your responsibility).

As most procedures are conducted during standard care, there will be no additional travel or participation compensation. You will be responsible for routine medical expenses, including surgery, exams, and follow-ups, as well as any unrelated medical costs.

9. Reasons for Study Withdrawal

You may be withdrawn from the study if:

- You do not comply with study requirements
- The investigator believes continued participation may pose a risk to you
- The study is discontinued
- Unforeseen circumstances arise

Even if you are withdrawn, routine follow-up for gastric cancer will continue.

10. Voluntary Participation and Withdrawal

Participation is entirely voluntary. Refusing or withdrawing from the study will not affect your medical care or treatment. Data collected up to the point of withdrawal may still be used for analysis. If new information arises that may affect your willingness to continue, it will be promptly shared with you or your legal representative.

11. Compensation and Medical Care in Case of Injury

We will make every effort to protect your safety during the study. The medications and procedures involved are standard treatments. If complications occur, appropriate medical care will be provided.

There is no financial compensation for anticipated side effects. However, the study is covered by insurance for unexpected serious injury, congenital disability, or death. If such an event occurs, you may contact the study team for assistance with the compensation process.

12. Privacy and Data Protection

As part of the study, we will collect data such as laboratory results, diagnostic and treatment records, and follow-up information. Personal identifiers (e.g., name, gender, contact details, hospital registration number) will also be collected for the purposes of monitoring and scheduling.

All data will be securely stored and encrypted, with access limited to authorized research staff. Regulatory agencies may review study records in accordance with applicable laws, and such access is permitted by your consent.

Study data will be stored for five years after completion and then securely destroyed. Your identity will not be disclosed in any reports or publications. By signing this form, you agree to the data collection and usage described above.

☐ I agree to the collection of my personal information. / ☐ I do not agree.

☐ I agree to the collection of my sensitive information. / ☐ I do not agree.

13. Study Contacts

If you or your legal representative have any questions or concerns about the study, please contact:

Principal Investigator:

Dr. In-Seob Lee

Professor

Division of Gastrointestinal Surgery

Department of Surgery

University of Ulsan College of Medicine and Asan Medical Center, Seoul, Korea

24-hour contact: +82-10-6595-0298

Study Nurses:

Eun-Young Jang, Ye-Eun Ryu

For questions about your rights as a research participant or to speak with someone not directly involved in the study:

Institutional Review Board (IRB): +82-2-3010-7166

Clinical Research Protection Center: +82-2-3010-7285

Clinical Study Participant Consent Form

Clinical Study Title:

Effect of Helicobacter pylori Eradication on Remnant Stomach Neoplasms after Curative Gastrectomy

1. I have received a verbal explanation of the study and have read the Participant Information Sheet. I have discussed the study with the responsible investigator.
2. I have been informed about the potential risks and benefits, and my questions have been answered to my satisfaction.
3. I voluntarily agree to participate in this study.
4. I understand that I may refuse to participate or withdraw from the study at any time without affecting my future medical care, and that such a decision will not result in any disadvantage to me.
5. By signing this information sheet and consent form, I agree to the collection and processing of my personal data by the researchers for medical research purposes, within the limits allowed by applicable laws and regulations.
6. I understand that I will receive a copy of this signed consent form.

Participant's Name: ____________________

Signature: __________________________

Date (YYYY/MM/DD): _________________

(If applicable)

Name of Legal Representative: ____________________

Signature: __________________________

Date (YYYY/MM/DD): _________________

Relationship to Participant: ______________________

(For example: parent of the patient)

Investigator's Name: ____________________

Signature: ____________________________

Date (YYYY/MM/DD): __________________

(If applicable)

Witness's Name: _______________________

Signature: ____________________________

Date (YYYY/MM/DD): __________________
