# Supplementary material for: Effect of Helicobacter pylori eradication on remnant stomach neoplasms after curative gastrectomy (HELP-GC): Protocol of a HELP-GC randomized controlled trial
Source: PLoS One. 2025 May 19;20(5):e0320903. doi: 10.1371/journal.pone.0320903 (PMC12088511; doi:10.1371/journal.pone.0320903)
Supplement: S2 Data — (PDF) [file pone.0320903.s004.PDF]

이 문서는 서울아산병원의 문서입니다. 동의없이 수정, 변경 및 복사할 수 없습니다.

1. Background

CRIS Registration Number	KCT0008855
Unique Protocol ID	2023-0660
Public/Brief Title	Effect of Helicobacter pylori eradication on remnant stomach neoplasm after curative gastrectomy
Scientific Title	Effect of Helicobacter pylori eradication on remnant stomach neoplasm after curative gastrectomy
Acronym	HELP-GC
MFDS Regulated Study	No
IND/IDE Protocol	No
Registered at Other Registry	No
Healthcare Benefit Approval Status	Submitted approval

2. Institutional Review Board / Ethics Committee

Board Approval Status	Submitted approval
Board Approval Number	2023-0835
Approval Date	2023-07-07
Approval File	IRB승인통지서.PDF
Institutional Review Board Name	Asan Medical Center Institutional Review Board
Institutional Review Board Address	(05505) Asan Medical Center Institutional Review Board, 88, Olympic-ro 43-gil, Songpa-gu, Seoul, Republic of Korea
Institutional Review Board Telephone	02-3010-7166
Data Monitoring Committee	Yes<br>Data Safety Monitoring Board

3. Contact Details

✓ Contact Person for Principal Investigator / Scientific Queries

Name	In-Seob Lee

이 문서는 서울아산병원의 문서입니다. 동의없이 수정, 변경 및 복사할 수 없습니다.

Title	Associate professor
Telephone	+82-2-3010-1728
Affiliation	Asan Medical Center
Address	(05505) Asan Medical Center, 88, Olympic-ro 43-gil, Songpa-gu, Seoul, Republic of Korea

✓ Contact Person for Public Queries

Name	Chang Seok Ko
Title	Clinical assistant professor
Telephone	+82-2-3010-0550
Affiliation	Asan Medical Center
Address	(05505) Asan Medical Center, 88, Olympic-ro 43-gil, Songpa-gu, Seoul, Republic of Korea

✓ Contact Person for Updating Information

Name	In-Seob Lee
Title	Associate professor
Telephone	+82-2-3010-1728
Affiliation	Asan Medical Center
Address	(05505) Asan Medical Center, 88, Olympic-ro 43-gil, Songpa-gu, Seoul, Republic of Korea

4. Status

Study Site	Single
Overall Recruitment Status	Recruiting
Date of First Enrollment	2024-06-27 Actual
Target Number of Participant	984
Primary Completion Date	
Study Completion Date	

✓ Recruitment Status by Participating Study Site 1

Name of Study	Asan Medical Center

이 문서는 서울아산병원의 문서입니다. 동의없이 수정, 변경 및 복사할 수 없습니다.

Recruitment Status	Recruiting
Date of First Enrollment	2024-06-27 ,

5. Source of Monetary / Material Support

✓ **1. Source of Monetary/Material Support**

Organization Name	Asan Medical Center
Organization Type	Medical Institute
Project ID	2022-00520

6. Sponsor Organization

✓ **1. Sponsor Organization**

Organization Name	Asan Medical Center
Organization Type	Medical Institute

7. Study Summary

Lay Summary	The causal relationship between Helicobacter pylori (H. pylori) infection and the development of gastric cancer is well established. Previous studies have reported a preventive effect of H. pylori (HP) eradication on the development of gastric cancer. Furthermore, the HP eradication (HPE) therapy successfully decreased the risk of recurrent gastric neoplasms after endoscopic resection. Contrary to the remarkable reduction in incidence of gastric neoplasm after endoscopic resection, there are few studies assessing the effect of HPE in condition of remained stomach after distal or subtotal gastrectomy. We aim to determine whether H. pylori eradication has preventive effect on the development of gastric neoplasm occurring in the remnant stomach after curative distal or proximal gastrectomy through a double-blinded, randomized controlled trial.

8. Study Design

Study Type	Interventional Study
Study Purpose	Treatment
Phase	Phase3
Intervention Model	Parallel
Blinding/Masking	Double

이 문서는 서울아산병원의 문서입니다. 동의없이 수정, 변경 및 복사할 수 없습니다.

Blinded Subject	Subject, Investigator
Allocation	RCT
Intervention Type	Drug
Intervention Description	1)Group A (Treatment arm)<br>Patients in the eradication arm receive the standard regimen consisting of proton pump inhibitor (esomeprazole 40 mg), amoxicillin 1 g, and clarithromycin 500 mg twice a day for 14 days<br>2)Group B (Placebo arm)<br>Patients in the control arm receive three placebo drugs.
Number of Arms	2
Arm 1 — Arm Label	Treatment arm
Arm 1 — Target Number of Participant	492
Arm 1 — Arm Type	Experimental
Arm 1 — Arm Description	1)Group A (Treatment arm)<br>Patients in the eradication arm receive the standard regimen consisting of proton pump inhibitor (esomeprazole 40 mg), amoxicillin 1 g, and clarithromycin 500 mg twice a day for 14 days<br>2)Group B (Placebo arm)<br>Patients in the control arm receive three placebo drugs.
Arm 2 — Arm Label	Placebo arm
Arm 2 — Target Number of Participant	492
Arm 2 — Arm Type	Placebo comparator
Arm 2 — Arm Description	2)Group B (Placebo arm)<br>Patients in the control arm receive three placebo drugs.

9. Subject Eligibility

Condition(s)/Problem(s)	* (C00-D48)Neoplasms<br>(C16.9)Malignant neoplasm of stomach, unspecified<br><br>Stomach Neoplasms Helicobacter
Rare Disease	No
Gender	Both
Age	19Year~70Year
Inclusion Criteria — Description	(1) aged 19–70 years<br>(2) who are diagnosed with H. pylori infection<br>(3) who are diagnosed with pathologic stage 1 gastric cancer<br>(4) who received distal gastrectomy for cancer located in the lower half of the stomach (pylorus, antrum, and lower body), and who received proximal gastrectomy for cancer located in the upper half of the stomach (fundus, cardia, upper body, and mid body) either requiring upfront curative gastrectomy or additional surgery after non-curative endoscopic resection<br>(5) not indicated to adjuvant chemotherapy
Exclusion Criteria	(1) history of HPE<br>(2) history of previous gastrectomy

이 문서는 서울아산병원의 문서입니다. 동의없이 수정, 변경 및 복사할 수 없습니다.

(3) history of any malignancy within recent 10 years
(4) Patient who previously undergone endoscopic treatment on the section that will become the remnant stomach
(5) patients who require neoadjuvant chemotherapy
(6) history of allergy or serious adverse events to prescribed medication including amoxicillin and clarithromycin
(7) presence of severe comorbidity (e.g. cardiac, hepatic, or renal insufficiency) or coagulopathy
(8) pregnant or lactating women
(9) presence of psychiatric disorder that may preclude compliance
(10) patients who cannot understand informed consent.
(11) Borrmann type 4 tumors (linitis plastica) on tumor classification
(12) Proximal resection margin shorter than 3cm in cases of advanced gastric cancer
(13) Declined to participate
(14) Withdrew consent

Healthy Volunteers

10. Outcome Measure(s)

Type of Primary Outcome	Not applicable

✓ **Primary Outcome(s) 1**

Outcome	The development of gastric neoplasms, including adenoma or adenocarcinoma in the remnant stomach of patients who received curative gastrectomy for gastric cancers
Timepoint	Upon the last enrollment

✓ **Secondary Outcome(s) 1**

Outcome	1) the 10-year overall survival, 2) the improvement rates of the gastric glandular atrophy and/or intestinal metaplasia, 3) HP eradication success rate, and 4) the incidence of new-onset hyperplastic polyps
Timepoint	Upon the last enrollment

11. Study Results and Publication

Result Registered	No

12. Sharing of Study Data(Deidentified Individual-Patient Data, IPD)

Sharing Statement	No
